# Quantum Approach for Contextual Search, Retrieval, and Ranking of Classical Information

**DOI:** 10.3390/e26100862

**Published:** 2024-10-13

**Authors:** Alexander P. Alodjants, Anna E. Avdyushina, Dmitriy V. Tsarev, Igor A. Bessmertny, Andrey Yu. Khrennikov

**Affiliations:** 1 Institute of Advanced Data Transfer Systems, ITMO University, 197101 St. Petersburg, Russia; 2 Faculty of Software Engineering and Computer Systems, ITMO University, 197101 St. Petersburg, Russia; avdiushina@itmo.ru (A.E.A.); bessmertny@itmo.ru (I.A.B.); 3 International Center for Mathematical Modeling in Physics, Engineering, Economics, and Cognitive Science, Linnaeus University, S-35195 Vaxjo, Sweden

**Keywords:** decision making, information retrieval, quantum cognition, ordering effect, machine learning

## Abstract

Quantum-inspired algorithms represent an important direction in modern software information technologies that use heuristic methods and approaches of quantum science. This work presents a quantum approach for document search, retrieval, and ranking based on the Bell-like test, which is well-known in quantum physics. We propose quantum probability theory in the hyperspace analog to language (HAL) framework exploiting a Hilbert space for word and document vector specification. The quantum approach allows for accounting for specific user preferences in different contexts. To verify the algorithm proposed, we use a dataset of synthetic advertising text documents from travel agencies generated by the OpenAI GPT-4 model. We show that the “entanglement” in two-word document search and retrieval can be recognized as the frequent occurrence of two words in incompatible query contexts. We have found that the user preferences and word ordering in the query play a significant role in relatively small sizes of the HAL window. The comparison with the cosine similarity metrics demonstrates the key advantages of our approach based on the user-enforced contextual and semantic relationships between words and not just their superficial occurrence in texts. Our approach to retrieving and ranking documents allows for the creation of new information search engines that require no resource-intensive deep machine learning algorithms.

## 1. Introduction

The exponentialgrowth of unstructured data currently imposes fundamentally new demands on information processing systems [1]. These requirements concern, firstly, the speed and, secondly, the quality of the processed information. Recently, quantum methods and approaches to information processing have become increasingly popular. Quantum approaches are quite understandable—they are applied to the development of effective quantum computers and simulators, ideally allowing for quantum speedup while solving NP-hard problems [2], including natural language processing [3,4]. As a result, the solution to these problems lies in the “bottleneck” of modern quantum technology development, which is expected to be significantly improved within the next 10–15 years, see e.g., [5]. This gives us hope for the eventual achievement of quantum supremacy.

On the other hand, the improvement of the quality of classical information processing and recognition by using quantum methods represents a complex interdisciplinary problem at the interfaces of computer science, artificial intelligence, quantum physics, and cognitive science [6,7]. Today, many areas apply quantum approaches in practice using the term “quantum-inspired”; however, this term has different meanings.

First, the quantum-inspired approach is applied to analog devices, which represent non-quantum hardware designed to enhance the performance of optimization problems [8]. Obviously, such systems cannot demonstrate quantum supremacy; however, their specific physical properties can accelerate the execution of some NP-hard algorithms.

Second, the quantum-inspired approach involves the development of probabilistic heuristic algorithms and relevant software that somehow simulate the behavior of quantum systems [9]. In this respect, the quantum-inspired approach includes quantum machine learning (QML) algorithms that combine quantum probabilistic approaches to information processing with classical machine learning methods [10]. In particular, the QML paradigm formulates an artificial intelligence agent’s (AIA) interaction with the environment within a quantum probability framework [11]. This approach opens a new direction for quantum-inspired algorithms that in some cases increase the efficiency and quality of classical information processing [6].

Third, the quantum-like modeling of natural intelligence agents (humans) is used in various tasks related to decision-making (DM) under conditions of uncertainty. Here, some questions arise: How relevant is the information to the user’s needs, and what are these needs that affect the user’s cognitive abilities, transformed by external informational “perturbations”? These issues play an important role in solving problems of social interaction in the economy, finance, social studies, etc. The way to solve them is to understand the fundamental properties of human perception, judgment, and decision-making. Recently, a phenomenon of the violation of classical probabilistic model predictions was obtained in practice, [12]. In particular, it is worth mentioning, as known in psychology, the question ordering effect, which presumes dependence of the (sequential) joint probability distribution of answers on the questions *A* and *B*; for the resulting probabilities $p_{AB}\neq p_{BA}$, see e.g., [13,14]. Quantum probability and measurement theory help to tackle this problem and obtain a reasonable explanation [15,16]. This is partly due to the complexity of the DM conditions themselves, the need to consider a large number of criteria [17], the “pressure” on the DM agent caused by large (excessive) information, and sometimes, on the contrary, its absence. In this case, the irrationality in DM caused by the influence of the DM agents’ psychology on these decisions establishes an important problem [18]. In particular, the works [12,19] drew attention to the fact that quantum probabilistic approaches may be useful for modeling judgments and decisions related to the so-called “cognitive biases” associated with irrational (contextually imposed) DM by a person under the condition of incomplete information [20].

It is interesting that many years ago, Niels Bohr pointed out the possibility of using the quantum apparatus of probability theory beyond physics, especially for tasks of perception, judgment, and DM in cognitive sciences [21]. Currently, a remarkable example of applying quantum probability beyond physics is the problem of retrieving and ranking information. Its purpose is to rank the documents that best match the user’s query. To solve this problem, all the power of quantum theory can be used: description of words and documents in a complex-valued Hilbert space, interference effects while searching and retrieving information, context-dependent measurement probabilities, and even entangled states, see [22]. In our view, some properties of quantum measurement theory can play an important role here, cf. [23].

In quantum science, accounting for the uncontrolled influence of the “apparatus” on the measured quantity [24] represents an important feature of the quantum approach to the measurement of some observable. Indeed, it is the source of various uncertainties in measured and conjugated observables. In quantum theory, observation probabilities of any quantity are always context-dependent and can change during the measurement. In this case, the retrieval problem represents the “interaction” of the user (human) with the computer, which can be considered as an “apparatus” for measuring the quantum-like cognitive state of the user in different contexts. The user’s cognitive state takes into account the uncertainty associated with the query. In many cases, the user does not know exactly how to phrase a query to obtain a uniquely satisfying answer. Furthermore, the user often does not know exactly what they are seeking. In this case, the user’s query represents a document measurement performed in some context. Quantum-like “interference” between document topics can change user preferences and requests during information retrieval. Namely, its state changes as a result of the back action of the “apparatus”, i.e., the computer that delivers the search result. Quantum probability theory, which depends on context, allows for taking all these factors into account relatively easily, without resorting to complex computational procedures based on deep machine learning techniques.

In this work, we consider a heuristic Bell-like algorithm that relates to the problem of document retrieving and ranking and is based on a two-word query given in different (mutually excluded) contexts. Our approach mimics the famous Bell test known in the framework of the Einstein–Podolsky–Rosen (EPR) paradox with the 1/2 spin of two particles in quantum science [25]. The main algorithm feature is the consideration of coupled queries in multiple document contexts. However, there are several important differences between the quantum physics approach and the quantum-like algorithm proposed below. First, in quantum physics, particles are indistinguishable. This is an important property of particle behavior that can be experimentally verified with entangled states, see e.g., [26]. Second, mathematically, the description of systems with *N* particles requires a $2^{N}$-dimensional Hilbert space and operates with tensors and complex structures based on them [27]. The usage of the full power of the Hilbert space does not make much practical sense in retrieving problems, since texts with a huge number of semantic units *N* occupy a large memory volume and will take a very long time to result on a classical computer. In this regard, our task here is to adapt the low-dimensional Hilbert space to classical information retrieving and ranking by using its useful algebraic constructions and properties, cf. [28,29].

There is a major difference between this work and the studies conducted in [28,29]. In this work, we take into account user preferences in different contexts and explore complex numbers specified in a Hilbert space for describing the user’s cognitive state. An additional phase parameter allows for taking into account the user’s attitude towards the different contexts in which queries are considered. We show that in this case, we need to use the full set of incompatible queries satisfying the SU(2) algebra. As a result, we obtain a ranking of documents more relevant to queries and user preferences.

## 2. Related Work

Recently, applying quantum probability theory, quantum structures, and quantum logic to the problem of natural language processing (NLP) has become of great interest, see e.g., [30,31,32]. Typically, the quantum approach generalizes formal (symbolic) [33] and distributive theories [34] in semantics. The latter account for the meaning of words that are usually represented as vectors inferred from the context [35]. Notably, the bag of words model, which accounts for the frequency of word occurrence in a document and frequency in different documents, represents a simple approach that can be used in NLP and IR [36]. However, the bag of words approach does not consider the word order, which is crucial for the text meaning in many languages. In the framework of semantic vector models, it is worth mentioning the latent semantic analysis (LSA) [37], hyperspace analogue to language (HAL) [38], and WORDSPACE [34], which require large word spaces. The distributive models in semantics, discussed in this work, represent areas of information retrieval in which quantum methods can be successfully implemented, cf. [39,40]. In particular, in [41], Zuccon et al. generalized the well-known probability ranking principle and formulated its quantum version, which evaluates the ranking criteria for documents and considers the dependencies between them. It is shown that quantum-like interference in document search and ranking plays an important role in the improvement of overall search efficiency.

The quantum-like approach to the problem of IR that we use below is based on the methods and approaches of distributive semantics, which determine the degree of semantic proximity between linguistic units in terms of their distribution in large data sets in the form of texts. In this case, words are represented by context vectors in a semantic Hilbert space H. The semantic proximity between words is defined by angles between the vectors. In a more general approach, we can assign vectors to individual documents in information retrieval and ranking problems. These documents can then be compared with the cosine measure of the proximity angle of documents. In this case, Hermitian operators correspond to the queries. Eigenvalues of these operators specify whether the object (document) is relevant or not [42,43]. The probability with which the eigenvalue of the query operator is obtained determines the degree of relevance of the word or some part of the document to a query.

## 3. Bell-like Test in Contextual Semantic Retrieving

### 3.1. Documents and Words as a Vectors in H

**Figure 1:**
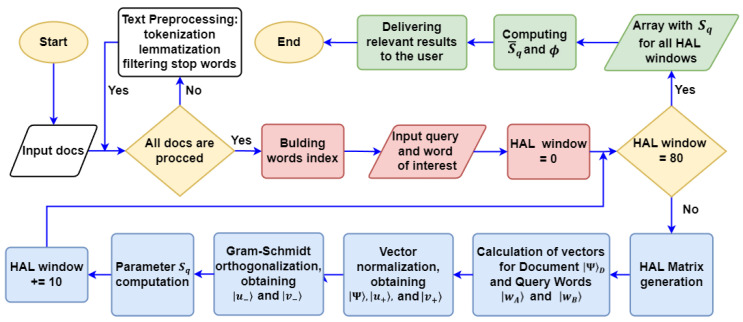
Timeline for a heuristic Bell-like algorithm for document retrieval.

The full timeline for suggested quantum-inspired algorithm implementation is presented in Figure 1. In this work, we employ the HAL approach to create a vector of words and documents specified in H; Algorithm S2 is schematically provided in Supplementary Materials, Section S1. In particular, we apply the HAL algorithm to a corpus of text, and this allows for using normalized strings of the HAL matrix as word context vectors to build a method for comparing text fragments, cf. Figure 1. The HAL matrix has dimension N×N, where *N* is the dictionary length. As a part of the HAL approach, a vector space of dimension *N* is constructed for the corpus of the text. Each word in this space is assigned to a vector; the normalized sum of such vectors forms the document for further processing. Each word obtains a coordinate not equal to zero if and only if somewhere in the text to the right of this word, in a limited window of length *d*, there is a word corresponding to this coordinate. The value of the assigned coordinate is inverse to the window distance to the word. Explicitly, if the word corresponding to the vector coordinate is *l* words away from the analyzed word, then the coordinate value of the HAL vector is L=d−l.

Thus, the HAL approach allows for finding the document state vector ${|\Psi \rangle }_{D}$ in the *N*-dimensional HAL space, see Figure 1. We suppose that ${|\Psi \rangle }_{D}$ extracted from the lines of the symmetric HAL matrix is

(1) $${|\Psi \rangle }_{D}=\sum_{i=1}^{N}|w_{i}\rangle ,$$

where $|w_{i}\rangle$ is *i*-th word vector. In this work, we restrict ourselves to two-word queries only. We examine how two query words *A* and *B* are relevant to each other within the document. We normalize ${|\Psi \rangle }_{D}$ and query word vectors $|w_{A}\rangle$ and $|w_{B}\rangle$, obtaining resulting document vector |Ψ〉 and normalized states $|u_{+}\rangle$ and $|v_{+}\rangle$, respectively. Then, applying the Gram–Schmidt orthogonalization procedure, we obtain another pair $|u_{-}\rangle$ and $|v_{-}\rangle$ of states orthogonal to $|u_{+}\rangle$ and $|v_{+}\rangle$, respectively. Algorithm S1 regarding the calculation is provided in the Supplementary Materials, Section S1.

Thus, we can decompose the resulting document vector |Ψ〉 as

(2a) $$\begin{array}{c} \begin{array}{c} \left|\Psi \rangle =a\right|u_{+}\rangle +b|u_{-}\rangle =\left(\begin{array}{c} a\\ b \end{array}\right), \end{array} \end{array}$$

(2b) $$\begin{array}{c} \begin{array}{c} \left|\Psi \rangle =c\right|v_{+}\rangle +d|v_{-}\rangle =\left(\begin{array}{c} c\\ d \end{array}\right). \end{array} \end{array}$$

**Figure 2:**
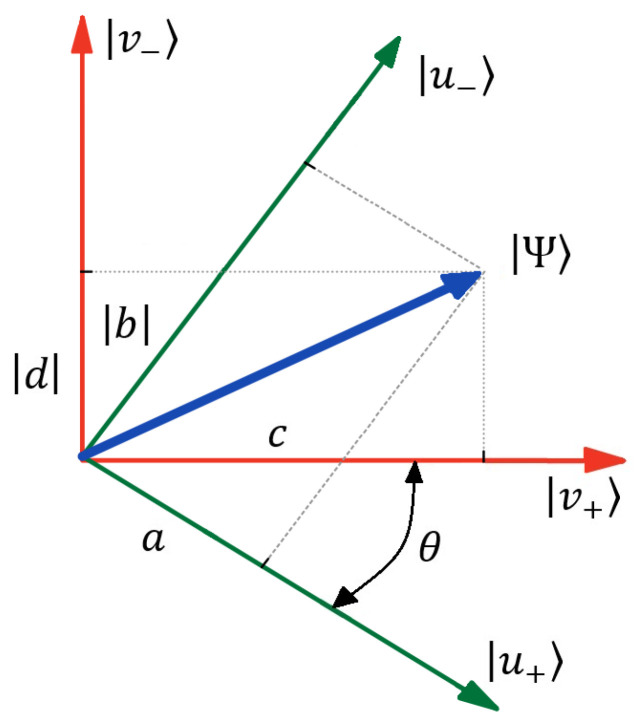
Schematic picture of the geometry of the vectors in a 2D Hilbert space with phase ϕ=0. Normalized document vector Ψ is decomposed into basis states $|u_{\pm }\rangle$, $|v_{\pm }\rangle$ of two query words, where indices + and − indicate complete relevancy and non-relevancy of the words to the query, respectively. θ is the angle between basis states. Other details are in the text.

We represent the geometric interpretation of (2) in Figure 2. In fact, the document state |Ψ〉 establishes as a qubit state represented in different frames. Basic states $|u_{+}\rangle$ and $|u_{-}\rangle$ can be recognized as the user’s cognitive states, in which they consider query word *A* to be fully relevant to the document or irrelevant, respectively. The same assumption is true for query word *B* operating within states $|v_{+}\rangle$ and $|v_{-}\rangle$. In quantum physics, states with “+” and “−” may be recognized as “spin-up” and “spin-down” states of 1/2 spin particles, respectively [25].

We define the projections of document vector |Ψ〉 onto the basic states from (2) as

(3a) $$\begin{array}{c} \begin{array}{c} a=\langle u_{+}|\Psi \rangle ,\;b=\langle u_{-}|\Psi \rangle , \end{array} \end{array}$$

(3b) $$\begin{array}{c} \begin{array}{c} c=\langle v_{+}|\Psi \rangle ,\;d=\langle v_{-}|\Psi \rangle . \end{array} \end{array}$$

In [28,29], it is assumed that all parameters *a*, *b*, *c*, and *d* specified in (3) are real. This case is displayed in Figure 2.

Probabilities $a^{2}$ and $c^{2}$ specify the relevance of one-word queries with words *A* and *B* to the document, separately. Notably, the coefficients in (3) obey normalization conditions

(4a) $$\begin{array}{c} \begin{array}{c} \;a^{2}+{\left|b\right|}^{2}=1, \end{array} \end{array}$$

(4b) $$\begin{array}{c} \begin{array}{c} \;c^{2}+{\left|d\right|}^{2}=1. \end{array} \end{array}$$

Then, matrix *M* defines transformation from the *A* to *B* bases

(5) $$M=\left(\begin{array}{cc} \langle v_{+}|u_{+}\rangle & \langle v_{+}|u_{-}\rangle \\ \langle v_{-}|u_{+}\rangle & \langle v_{-}|u_{-}\rangle \end{array}\right)=\left(\begin{array}{cc} p & \sqrt{1-p^{2}}\\ -\sqrt{1-p^{2}} & p \end{array}\right),$$

where $p=\langle v_{+}|u_{+}\rangle =\cos\left(\theta \right)$ characterizes the angle between word vectors $|u_{+}\rangle$ and $|v_{+}\rangle$ in a Hilbert space, see Figure 2 cf. [28]. *p* defines the contextual proximity of semantic concepts as a function of θ. We can recognize $p^{2}$ as a probability of any selected words appearing together in a chosen document. In particular, the limiting value, p=1, establishes a case in which two arbitrary words in the text are very close to each other contextually or even coincide. On the contrary, p=0 specifies the case when two words are well resolved in meaning and context; the word vectors are orthogonal to each other.

This work examines the case when $b=\left|b\right|e^{i\phi_{b}}$, $d=\left|d\right|e^{i\phi_{d}}$ are complex numbers. The phase factors, $\phi_{b,d}$, represent additional parameters for the discussed quantum-inspired algorithm of information retrieving, cf. [29]. We recognize $\phi_{b,d}$ as parameters that specify user (cognitive) initial preferences within information retrieval and document ranking in respect to two words. Notice that without any special preferences, we can put formally $\phi_{b,d}=0$ in (4), and real projections b=|b|, d=|d| are shown in Figure 2.

Notably, the phases θ, $\phi_{b,d}$ in the framework of quantum probability theory specify two types of interference effects that occur in the presence of “interaction” of the user with the computer as an “apparatus” for measuring the quantum-like cognitive state of the user in different contexts. In particular, in mathematical formalism, the searcher’s preference can be coupled to interference between the search for words *A* and *B*. In the quantum formalism, interference is manifested as interference of probabilities, and the phase parameter θ quantifies the degree of interference and deviation from the classical probability theory [44]. Feynman suggested coupling interference with violation of additivity of probability [45,46]. In [47], Feynman’s reasoning was reformulated as a violation of the formula of total probability (FTP). For two arbitrary dichotomous observables $a=\alpha_{1},\alpha_{2}$ and $b=\beta_{1},\beta_{2}$, FTP with the interference term has the form

(6) $$P\left(b=\beta \right)=\sum_{\alpha }P\left(a=\alpha \right)P\left(b=\beta |a=\alpha \right)$$

$$+2\sum_{\alpha_{1}<\alpha_{2}}\cos\theta_{\alpha_{1}\alpha_{2}}\sqrt{P\left(a=\alpha_{1}\right)P\left(b=\beta |a=\alpha_{1}\right)P\left(a=\alpha_{2}\right)P\left(b=\beta |a=\alpha_{2}\right)}$$

The proof of this formula is based on the representation via squared absolute values of the corresponding complex amplitudes and the projection postulate for update of these amplitudes and, hence, the probabilities; for its application to cognitive science and decision making see, e.g., [48]. The phase $\phi_{b}=\phi_{d}=\phi$ that we introduce below (see (17)) specifies interference that accounts for user preferences in respect to a two-word query determined by their cognitive state in general. Pragmatically, one can treat ϕ as just an additional parameter to model preferences. This parameter is consistently transformed within the complex Hilbert space calculus for quantum probabilities.

#### One-Word Queries

It is necessary to specify query operators that act on the states of words and document vectors in H. First, we define the operators for the *A* word, which specifies the query in the primary context for the user. In particular, operator $A_{z}$ has two eigenvalues +1 and −1 in the word bases $|u_{\pm }\rangle$, respectively. The complete set of query operators, $A_{j}$ (j=x,y,z) for the *A* word operating in H, obeys SU(2) algebra symmetry properties and may be established by the Pauli matrices as

(7a) $$\begin{array}{c} \begin{array}{c} \;A_{x}=\left(\begin{array}{cc} 0 & 1\\ 1 & 0 \end{array}\right); \end{array} \end{array}$$

(7b) $$\begin{array}{c} \begin{array}{c} \;A_{y}=\left(\begin{array}{cc} 0 & -i\\ i & 0 \end{array}\right); \end{array} \end{array}$$

(7c) $$\begin{array}{c} \begin{array}{c} \;A_{z}=\left(\begin{array}{cc} 1 & 0\\ 0 & -1 \end{array}\right). \end{array} \end{array}$$

Notice that we can recognize non-commuting operators (7) as mutually incompatible sets of queries relevant to different contexts of the word. Practically, operators $A_{x}$ and $A_{y}$ may be associated with some other (even initially unknown) contexts of work *A*, which is incompatible with the $A_{z}$ query.

We can define the query operators for the second word, *B*, by using unitary transformation $B_{j}=M^{-1}A_{j}M$, j=x,y,z, where $M^{-1}$ is inverse to the *M* matrix defined in (5):

(8a) $$\begin{array}{c} \begin{array}{c} \;B_{x}=\left(\begin{array}{cc} -2p\sqrt{1-p^{2}} & 2p^{2}-1\\ 2p^{2}-1 & 2p\sqrt{1-p^{2}} \end{array}\right); \end{array} \end{array}$$

(8b) $$\begin{array}{c} \begin{array}{c} \;B_{y}=\left(\begin{array}{cc} 0 & -i\\ i & 0 \end{array}\right); \end{array} \end{array}$$

(8c) $$\begin{array}{c} \begin{array}{c} \;B_{z}=\left(\begin{array}{cc} 2p^{2}-1 & 2p\sqrt{1-p^{2}}\\ 2p\sqrt{1-p^{2}} & 1-2p^{2} \end{array}\right). \end{array} \end{array}$$

As seen from (5)–(8), our approach leaves transfer matrix *M* and word query operators $A_{x,y,z}$, $B_{x,y,z}$ independent on $\phi_{b,d}$. In this work, we restrict ourselves to the case when the user initially does not have certain preferences between two-word queries. However, such preferences may arise at the stage of document relevance assessment. Thus, average values of operators in (7), (8) specify the relevance degree of the words *A* and *B* to queries, respectively. To be more specific, we use Equation (2a) for document state |Ψ〉 representation when averaging query operators. At this stage of algorithm implementation, we assume that the *b* parameter in (2a) is a complex number that possesses phase $\phi \equiv \phi_{b}$.

Using the quantum state of the document, |Ψ〉, and query operators (7), we can determine document-averaged query values for word *A* in different (mutually “orthogonal”) contexts. In particular, average values $\langle A_{x,y,z}\rangle$ look like

(9a) $$\begin{array}{c} \begin{array}{c} \langle A_{x}\rangle =2a\sqrt{1-a^{2}}\cos\left(\phi \right); \end{array} \end{array}$$

(9b) $$\begin{array}{c} \begin{array}{c} \langle A_{y}\rangle =2a\sqrt{1-a^{2}}\sin\left(\phi \right); \end{array} \end{array}$$

(9c) $$\begin{array}{c} \begin{array}{c} \langle A_{z}\rangle =2a^{2}-1. \end{array} \end{array}$$

Notably, as follows from (9), the value of $\langle A_{z}\rangle$ is independent of user preferences (ϕ parameter) among the three (basic) queries.

Analogously, averaging Equation (8) over the state of the document, |Ψ〉, we obtain

(10a) $$\begin{array}{c} \begin{array}{c} \langle B_{x}\rangle =2\left(1-2a^{2}\right)p\sqrt{1-p^{2}}+2a\sqrt{1-a^{2}}\left(2p^{2}-1\right)\cos\left(\phi \right); \end{array} \end{array}$$

(10b) $$\begin{array}{c} \begin{array}{c} \langle B_{y}\rangle =2a\sqrt{1-a^{2}}\sin\left(\phi \right); \end{array} \end{array}$$

(10c) $$\begin{array}{c} \begin{array}{c} \langle B_{z}\rangle =\left(2a^{2}-1\right)\left(2p^{2}-1\right)+4a\sqrt{1-a^{2}}p\sqrt{1-p^{2}}\cos\left(\phi \right). \end{array} \end{array}$$

**Figure 3:**
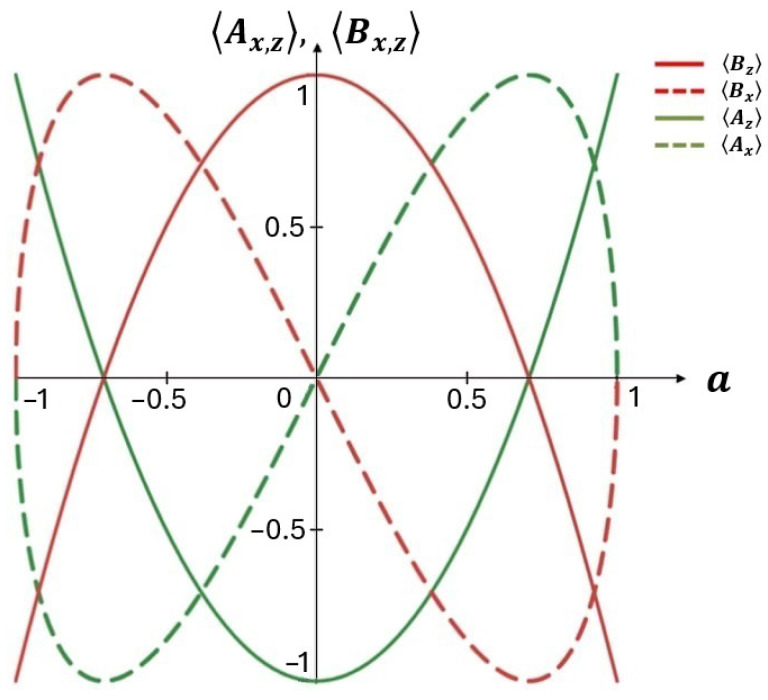
Average values of query operators $\langle A_{x,y}\rangle$, $\langle B_{x,y}\rangle$ vs. *a* for p=0 (θ=π/2).

In Figure 3 we plot the average values of query operators $\langle A_{x,z}\rangle$ and $\langle B_{x,z}\rangle$ as functions of real parameter *a* and without user preferences, ϕ=0. In this case, $\langle A_{y}\rangle =\langle B_{y}\rangle =0$, see (9), (10). For Figure 3, we choose the case when two query vectors are mutually orthogonal. In particular, as seen from Figure 3, word *A* (word *B*) is maximally relevant, $\langle A_{z}\rangle =1$, (irrelevant, $\langle B_{z}\rangle =-1$) to the document at a=1. At a=0 the opposite case ($\langle A_{z}\rangle =-1$, $\langle B_{z}\rangle =1$ ) is observed in Figure 3. However, at $a=\sqrt{0.5}≃0.71$ we can obtain $\langle A_{z}\rangle =\langle B_{z}\rangle =0$, which manifests (semantic) uncertainty in the chosen context of two-word queries. In this case, the degrees of relevance for queries $\langle A_{x}\rangle$ and $\langle B_{x}\rangle$ are maximal and minimal, respectively.

**Figure 4:**
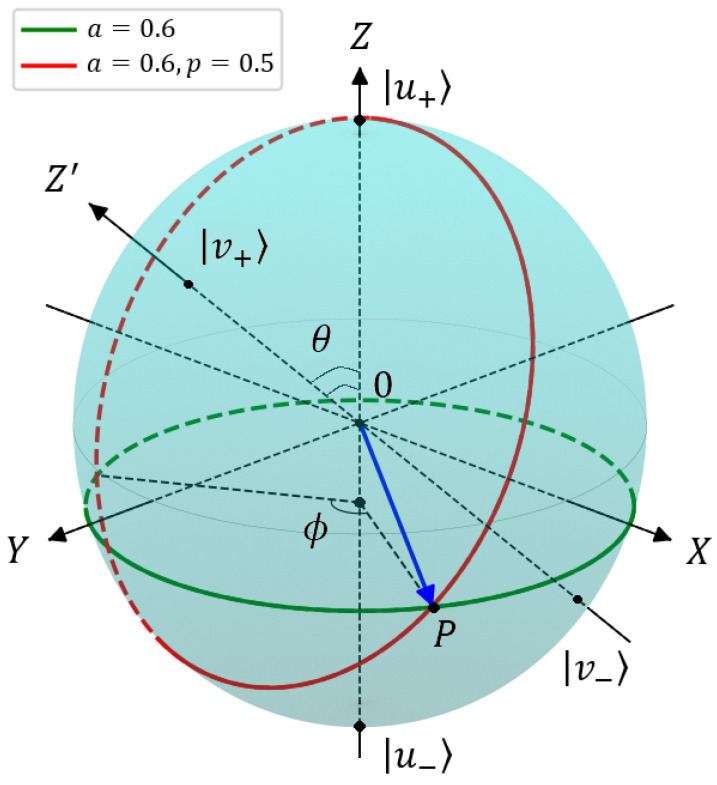
Geometrical representation of query relevance in the Bloch sphere. The details are given in the text.

In the presence of user interests (ϕ≠0), it is informative to represent average values (9), (10) on the Bloch sphere, see Figure 4. The north pole in Figure 4 corresponds to the maximum value of $\langle A_{z}\rangle =1$ (a=1) that establishes the full relevance of the document to the query based on the word *A*. The south pole in Figure 4 corresponds to the minimum value of $\langle A_{z}\rangle =-1$ (a=0), indicating that the document is completely irrelevant to the query. The green circle-like trajectory in Figure 4 reflects the uncertainty of the relevance evoked by an infinite number of contexts the user is interested in for query word *A*, which is described by phase parameter 0≤ϕ≤2π. Any point on the Bloch sphere in Figure 4 is determined by given *a* and chosen ϕ in Equation (9). Thus, the points on the Bloch sphere reflect certain user preferences in average relevances of queries $A_{x,y,z}$; in Figure 4, we present point *P* as an example.

The red line in Figure 4 represents a plot of Bloch vector components $\langle B_{j}\rangle$ for the relevances of second word *B* average queries. Angle θ between two words can be represented now as the angle between axes 0Z and $0Z^{\prime }$. It follows from (10) and Figure 4 that if two words coincide in meaning and context, then p=1, and $\langle B_{z}\rangle =\langle A_{z}\rangle$. Otherwise, for p=0, we have $\langle B_{z}\rangle =-\left(2a^{2}-1\right)=-\langle A_{z}\rangle$. The most distinguishable words are determined by angle θ=π/2 between the word vectors; in this limit, we can assume p=0 in (10).

### 3.2. Two-Word Queries, Bell-like Test Heuristic Algorithm

We can express the query that contains two words, *A* and *B*, simultaneously in terms of Bell-like parameter $S_{q}$ defined as (cf. [49])

(11) $$S_{q}=\left|\langle A_{z}B_{0}\rangle +\langle A_{x}B_{0}\rangle +\langle A_{z}B_{1}\rangle -\langle A_{x}B_{1}\rangle \right|,$$

where $B_{0}$ and $B_{1}$ are specified as

(12a) $$\begin{array}{c} \begin{array}{c} B_{0}=-\frac{1}{\sqrt{2}}B_{x}-\frac{1}{\sqrt{2}}B_{z}; \end{array} \end{array}$$

(12b) $$\begin{array}{c} \begin{array}{c} B_{1}=\frac{1}{\sqrt{2}}B_{x}-\frac{1}{\sqrt{2}}B_{z}. \end{array} \end{array}$$

Equation (12) allows for representing parameter $S_{q}$ as

(13) $$S_{q}=\sqrt{2}\left|\langle A_{z}B_{z}\rangle +\langle A_{x}B_{x}\rangle \right|.$$

From (13), it is seen that maximal value $S_{q}=2\sqrt{2}$, occurs for $\langle A_{z}B_{z}\rangle =\langle A_{x}B_{x}\rangle$; in quantum theory, this defines the so-called upper (Tsirelson’s) bound [50]. In the framework of the EPR paradox, we deal with two indistinguishable particles in $2^{2}$D Hilbert space; Equations (11) and (13) specify the Bell-type measurements of particles’ spin components in mutually incompatible *z* and *x* bases [25]. The true (quantum) entanglement between particles presumes the existence of their joint (non-separable) wave function, which causes a violation of the so-called Clauser–Horne–Shimony–Holt (CHSH) inequality, cf. [49]. Notice that operators of observables in this case must satisfy certain commutation relations, see, e.g., [51,52].

In the examples considered below, two words are “distinguishable” ab initio; we project the document state onto the 2D Hilbert space and examine word *B* query in the framework of word *A*, which leads to the non-equivalence of queries AB and BA, respectively. Mathematically, we are able to rely here on non-commuting operators in (13), which follows from the definitions (7), (8), i.e.,

(14) $$\left[A_{x},B_{x}\right]=\left[A_{z},B_{z}\right]=4ip\sqrt{1-p^{2}}A_{y},$$

where $A_{y}=B_{y}$, see (7), (8). Equation (14) manifests the incompatibility of word combination AB and BA queries.

We emphasize that the non-equivalence property of the orders AB and BA fits well with the well-known order influence of decision theory. The recognition of words plays the role of questions that are asked sequentially. In decision theory, non-coincidence of probabilities $p_{AB}$ and $p_{BA}$ was modeled in the quantum-like framework by exploiting the non-commutativity of operators representing the questions *A* and *B*, cf. [53,54]. Later, more general quantum-like models within the theory of quantum instruments were designed [16]. Thus, our model is a natural extension of studies on the order effect in cognitive psychology and social science. As well as in decision-making, the use of quantum instruments may improve the model, but this is a topic for further studies.

Notice that symmetrizing the definition of parameter $S_{q}$ in (13) relative to the sequence of words in two-word queries does not make much practical sense since the user in real situations makes queries of words in a certain sequence, which already provides a specific user context and preferences. The quantum approach, which uses the HAL method and takes into account the non-commutative nature of AB, BA queries, seems very reasonable for the problem of ranking documents. Let us consider simple examples. Obviously, the query *“Fish Food”* and *“Food Fish”* will appear with completely different meanings and contexts in the texts. The order *“Fish Food”* is about food for fish. As expected, the context of this query assumes that the user is looking for food for aquarium fish. The query *“Food Fish”* assumes a context such that the user is looking for fish as food for human consumption. Obviously, the results of the AB and BA requests in this case will be completely different. The HAL approach distinguishes such queries because it takes into account the context of the words in the text. In particular, in our HAL model, the context is constructed dynamically, as the window size influences the weighting of nearby words, making the representation of the query context more flexible compared to static models. The family of retrieval methods based on the bag-of-words approach cannot do this because it only considers the frequency of occurrence of words in the text, cf. [55]. In this paper, we examine an example of AB and BA queries that may be more relevant to real-world situations. In particular, we consider queries for a combination of two words AB (*“Summer Tour”*) and BA (*“Tour Summer”*), where the context in which they appear in the document is important for the user. To be more specific, we represent this context with additional words *“Active”* (which specifies the user wishes for outdoor activities) and *“Beach”* (which presumes sunbathing on the beach, for example), respectively. In the following, we analyze in detail how queries for two words in different sequences lead to (slightly) different document ranking results.

Notably, here we cannot talk about the effects of true quantum entanglement. The “entanglement” between two words can be interpreted as a pair of words that appear simultaneously in the text within different contexts (in quantum physics, this corresponds to different polarization basis, cf. [25]). Furthermore, “entanglement” in our approach is reflected in the interdependence of word pairs across different contexts, where changing one query word affects the retrieval relevance of the whole phrase, much like quantum states affect each other. This context-dependent behavior simulates quantum-like entanglement in semantic space. The Bell-like parameter $S_{q}$ in this case characterizes some uncertainties that appear within the human–computer interactions for document retrieval in different contexts that are based on certain algebraic properties of the query operators (7) and (8).

Using (2)–(5), from Equations (7) and (8) for (13), we obtain

(15) $$S_{q}=2\sqrt{2}\sqrt{{\left(-1+2p^{2}\right)}^{2}+16p^{2}\left(1-p^{2}\right)a^{2}\left(1-a^{2}\right)\sin^{2}\left(\phi \right)}.$$

Equation (15) represents one of the important results of this work. At ϕ=0, from (15) we obtain

(16) $$S_{q}=2\sqrt{2}\left|-1+2p^{2}\right|.$$

Equation (16) is reminiscent of the results obtained in [28,29]. In fact, in this limit, we deal with the Hilbert space that exploits all really defined vectors, see (2), (3).

**Figure 5:**
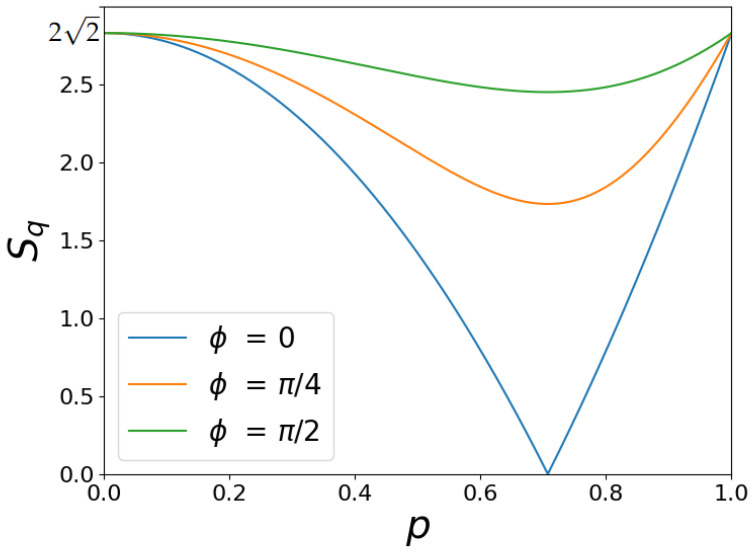
Bell-like parameter $S_{q}$ dependencies on *p*.

In Figure 5, we plot the dependency of Bell-like parameter $S_{q}$ on *p* for different values of ϕ. Remarkably, $S_{q}=2\sqrt{2}$ at p=0 and p=1, respectively. As can be seen from Figure 3, in this case, the average values of word queries $\langle A_{z}\rangle ,\langle B_{z}\rangle$ achieve their extreme values $\langle A_{z}\rangle =-1$, $\langle B_{z}\rangle =1$ and $\langle A_{z}\rangle =-1$, $\langle B_{z}\rangle =1$, respectively. In particular, the case p=0 is reminiscent of the maximally entangled two-particle state $|\Psi \rangle =\frac{1}{\sqrt{2}}{(|↑\rangle }_{A}{|↓\rangle }_{B}+{|↓\rangle }_{A}{|↑\rangle }_{B})$ that occurs for two oppositely aligned spins; in our case, this situation corresponds exactly to the case of angle θ=0 between the *A* and *B* words. Another value p=1 establishes entangled state $|\Psi \rangle =\frac{1}{\sqrt{2}}{(|↑\rangle }_{A}{|↑\rangle }_{B}+{|↓\rangle }_{A}{|↓\rangle }_{B})$.

Interestingly, parameter $S_{q}$ in Figure 5 decreases in range 0<p<1. Practically, it can be associated with the increase of semantic uncertainty in queries when the angle between words *A* and *B* varies in the range of 0<θ<π/2. In particular, $S_{q}$ in Figure 5 vanishes at $p=\sqrt{0.5}$ (θ=π/4). In this limit, average queries $\langle A_{z}\rangle ,\langle B_{z}\rangle$ related to the primary context remain maximally semantically uncertain at ϕ=0, i.e., $\langle A_{z}\rangle =\langle B_{z}\rangle =0$. However, the situation changes if we account for certain user preferences in terms of other contexts. The orange and green curves in Figure 5 express the user’s awareness of other (non-primary) query contexts. Thus, we can conclude that the Bell-like parameter $S_{q}$ provides reasonable information for the relevance of two-word queries in the presence of certain user preferences.

Finally, it is important to note that the obtained Equations (14)–(16) and relevant dependencies in Figure 5 look independent of HAL window size. However, as shown below, the $S_{q}$ parameter is sensitive to the HAL window. We suspect that the size of the HAL window is similar to some properties of detectors that count photon coincidences in the experiment with EPR pairs. In particular, statistical properties of photon detection depend on the detector’s response time, photon observation time, etc.; see, e.g., [56].

## 4. Results

### 4.1. Quantum-Inspired Algorithm Based on the Bell-like Test

The Bell-like test we discuss in this work represents the core of our heuristic quantum-inspired document ranking algorithm that accounts for word contexts and user preferences. The concrete steps for algorithm processing and verification are established below and given in Figure 1.

#### 4.1.1. Dataset Preparation

To implement the first part of the algorithm (white boxes in Figure 1), we use a dataset of synthetic advertising text documents from travel agencies. Namely, we examine eight texts describing services and offers in the field of tourism; the titles of the documents studied are listed in Table 1. The full texts of documents generated by the *OpenAI* **GPT-4** model are presented in Supplementary Materials (Section S2).

The following text generation parameters were used in the experiments:

- Temperature (0.7), which determines the degree of randomness when choosing words.
- Top-p (0.9), which limits the choice of the following words to probabilities that form a total probability of 0.9.

The texts were generated under controlled conditions without using any additional restrictions on the frequency and presence of words in order to exclude the influence of external modifications on the analysis results. Each text was tokenized using the **spaCy** library, allowing the text to be split into separate tokens (words and punctuation). Then, we performed lemmatization with **spaCy**, which ensured that words were reduced to their basic forms, improving the accuracy of the subsequent text analysis. Based on the lemmas obtained, an index was created for each document. In addition, a HAL matrix was created for each document and was then used to calculate values within the Bell-like test. Each word in the text was transformed into a vector with the HAL algorithm; see rose boxes in Figure 1.

#### 4.1.2. User Preferences Evaluation

The ϕ phase, which reflects user preferences, is calculated as the angle between the user’s interest vector and the document vector in the Hilbert space, providing not only the evaluation of explicit semantic relationships between the user’s query and the document content but also the identification of hidden similarities. For practical purposes, we specify the phase as

(17) $$\phi =\arccos\left[\frac{\left|\langle \Phi |\Psi \rangle \right|}{\sqrt{\langle \Psi |\Psi \rangle \langle \Phi |\Phi \rangle }}\right],$$

where |Φ〉 is the vector of user preferences in respect to a two-words query. Strictly speaking, definition (17) depends on the word sequence in the query. In practice, |Φ〉 might be obtained by applying machine learning techniques for search engines to investigate whether users have certain preferences. Furthermore, as |Φ〉, we use vectors of preselected words that determine the key interests of a given user. As a result, knowing states |Φ〉 and |Ψ〉, we can calculate metrics $S_{q}$ for different HAL window sizes, see (15).

#### 4.1.3. Bell-Like Parameter Verification

**Figure 6:**
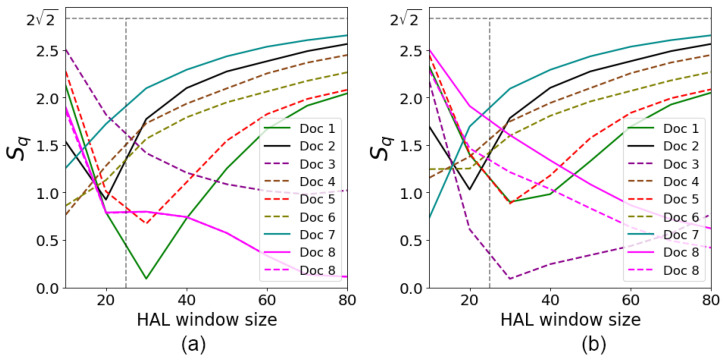
Bell-like parameter $S_{q}$ dependencies on the size of the HAL window at ϕ≠0 for the user queries (**a**) *“Summer Tour”* and (**b**) *“Tour Summer”*. The documents are taken from Table 1. The vertical dashed line corresponds to the window size equal to 25. The horizontal gray dashed line $S_{q}=2\sqrt{2}$ indicates a maximal value for document ranking. User interests *“Active”* and *“Beach”* are marked with the color solid and dashed curves, respectively.

**Figure 7:**
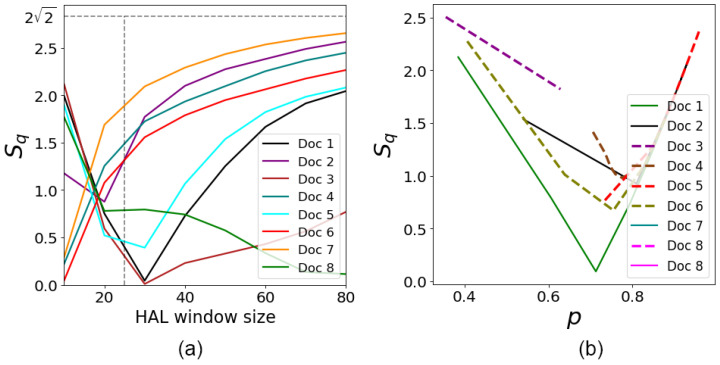
(**a**) Bell-like parameter $S_{q}$ versus the size of the HAL window at ϕ=0 for user query *“Summer Tour”*, cf. Figure 6. The results for user query *“Tour Summer”* are the same. (**b**) Dependencies of $S_{q}$ on *p* for the curves shown in Figure 6.

**Figure 8:**
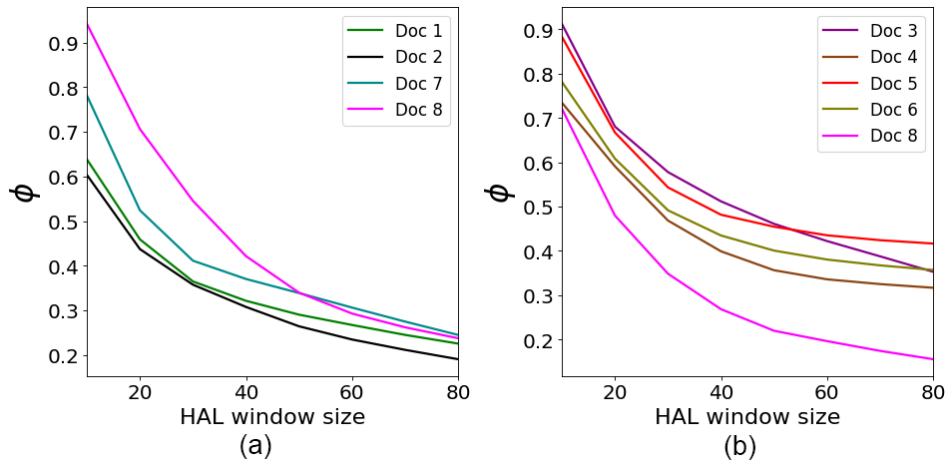
The ϕ phase vs. HAL window size for the user preferences (**a**) *“Active”* and (**b**) *“Beach”*.

Thus, based on the processed text data of the documents listed in Table 1, we aimed to find the relationships between Bell-like parameter $S_{q}$, the probability $p^{2}$ of two words occurring together in the chosen document, and phase ϕ at different HAL window sizes. The remarkable features of these parameters are presented in Figure 6, Figure 7b, and Figure 8, respectively.

Figure 6 demonstrates the dependence of $S_{q}$ on the HAL window size obtained with two different interests |Φ〉 *“Active”* (solid curves) and *“Beach”* (dashed curves) of the user. In particular, we examine different sequences of the words in Figure 6; the words *“Summer Tour”* were used as the user query for Figure 6a and *“Tour Summer”* for Figure 6b.

Figure 6 exhibits document ranking at the given size of the HAL window. In particular, for Figure 6a and Figure 6b, we plot two gray dashed vertical lines that correspond to the HAL window size equal to 25, respectively. The points where the curves in Figure 6 cross these vertical lines establish the document ranking for the selected size of the HAL window. It is remarkable that the behavior of $S_{q}$ in Figure 6 indicates dependency on *p*, cf. Figure 5.

To be more specific, let us analyze the document retrieval and ranking shown in Figure 6a. It illustrates that the curves for the interest *“Active”* start lower in most cases and grow faster compared to the curves for the *“Beach”* interest. This may indicate that the documents corresponding to outdoor activities are less context-sensitive at smaller HAL window sizes but that they become more relevant as the context increases. For example, routes in Patagonia and cycling tours in Tuscany show a high proximity to the “Active” interest, which is obvious given their active and adventurous nature. Figure 6a demonstrates that Document 8 is equally relevant for both queries and that the $S_{q}$ value drops as the window increases. Considering some of the occurrences of query words in the text, the word *“Active”* can have a strong impact on the initial smaller HAL windows, especially if it is central to other keywords in the document. This creates denser and more meaningful semantic connections. As the window size increases, the relationship between *“Active”* and other words such as *“Tour”*, *“Summer”*, and *“Beach”* becomes less pronounced. This is due to the likelihood of *“Active”* encountering each of these words as the window decreases. For the interest *“Active”*, the high frequency of this word’s presence in small windows contributes to a better match of interests and content. However, as the window increases, this match decreases. For *“Beach”*, references to this word do not create strong enough contextual links with other keywords to keep $S_{q}$ stable, especially when the window enlarges.

Figure 6b demonstrates some differences in the behavior of curves when we change the sequence of words in the two-word query. Two important properties of such changes are significant here.

First, the changes are visible at relatively small HAL window sizes. We can recognize this behavior as a “quantum size” effect, which is directly related to the quantum approach to document ranking. For large HAL window sizes, the asymptotic behavior of the curves in Figure 6a and Figure 6b exhibits similar characteristics.

Second, the behavior of the curves in Figure 6a and Figure 6b is significantly different only for some documents. In particular, for Document 3, the association between the words *“Active”* and *“Tour”* is significantly stronger due to their frequent co-occurrence within the same context, which enhances their frequency, making the document more sensitive to the order of words. Document 8 is likely focused on beach destinations or beach vacations, rendering its response curve more sensitive to queries where *“Summer”* is emphasized as the primary element.

Since Document 8 matches the interests in both *“Active”* and *“Beach”*, the context, in which the words meet, plays a crucial role. Thus, while the curves align in query *“Tour Summer”*, they diverge dramatically in *“Summer Tour”* because the context of *“Summer”* and *“Active”* does not match that of *“Summer”* and *“Beach”*. Notably, the frequency plot also shows an uneven distribution of words, Notably, the frequency plot that we examine below, also shows an uneven distribution of words highlighting how the semantic context influences the document’s sensitivity to different queries, highlighting how the semantic context influences the document’s sensitivity to different queries.

Figure 7a shows the results of the Bell-like parameter simulation for the documents analyzed under condition ϕ=0. This condition corresponds to the case when the user’s interests do not contribute to the evaluation of the document’s relevance, which makes it possible to observe a purely objective semantic interaction of words in the document based solely on their contextual connectivity. Notably, at ϕ=0, the ranking results for queries *“Summer Tour”* and *“Tour Summer”* match. This property can be used to evaluate the impact of the user’s preferences.

The important features of the proposed algorithm follow from Figure 7b and Figure 8. Figure 7b illustrates dependencies of $S_{q}$ on *p* of the words occurring together for the eight documents. Each curve in Figure 7b represents the document from Table 1 and the corresponding curve in Figure 6. The plots in Figure 7b clearly exhibit the dependencies for the Bell-like parameter obtained theoretically in (15) and shown in Figure 5.

Figure 8 demonstrates the dependence of phase angle ϕ on the size of the HAL window for the documents taken from Table 1 and classified by user preferences. As expected, the value of ϕ drops as the HAL window size increases. This behavior indicates that the influence of user preferences on document ranking decreases as the context window size grows, containing more words of the text. As seen from Figure 8, the curves representing the *“Beach”* preference show a slower decrease in ϕ. This may indicate a weaker word connectivity in these documents.

Notably, from the practical point of view, it is fruitful to examine the average variable, ${\bar{S}}_{q}$, that considers parameter $S_{q}$ averaged over all HAL window sizes. This allows us to select only the robust contextual relationships between the words of the document and the query. We define the average variable as

(18) $${\bar{S}}_{q}=\frac{1}{W}\sum_{i=1}^{W}S_{q,i},$$

where *W* is the number of windows of different sizes. We assume that the ${\bar{S}}_{q}$ parameter representation provides practical results for document retrieval and a ranking algorithm that is independent of particular HAL window sizes, see Figure 1.

#### 4.1.4. Cosine Similarity Metrics

The efficiency of the proposed approach was compared with the cosine similarity calculated using TF–IDF vectorization, cf. [57]. This approach is widely used to evaluate the closeness of document text to user queries. It does not only consider the frequency of each word in the document (term frequency, TF) but also the inverse frequency of documents containing that word (inverse document frequency, IDF). Thus, the TF–IDF metric, *T*, for a given word, *A*, in the document, *D*, can be expressed as

(19) $$T=\frac{n_{A,D}}{n_{D}}\log\left[\frac{N}{N_{A}}\right],$$

where $n_{A,D}$ is the number of occurrences of word *A* in document *D*; $n_{D}$ is the total number of words in document *D*; *N* is the total number of documents; and $N_{A}$ is the number of documents containing word *A*.

Then, the cosine similarity can be calculated using the obtained TF–IDF vectors for documents and user queries:

(20) $$S_{\cos}=\frac{\langle \psi_{Q}|\Psi \rangle }{\sqrt{\langle \Psi |\Psi \rangle \langle \psi_{Q}|\psi_{Q}\rangle }},$$

where $|\psi_{Q}\rangle$ is the user query vector. It should be noted that the cosine similarity (20) depends on the frequency of keywords in the text and their importance, which makes it sensitive to the lexical content of the document. On the other hand, the Bell-like metric $S_{q}$ is based on the analysis of contextual relationships and is resistant to changes in the analysis parameters. This allows for considering deeper semantic relationships between the words.

## 5. Discussion

**Figure 9:**
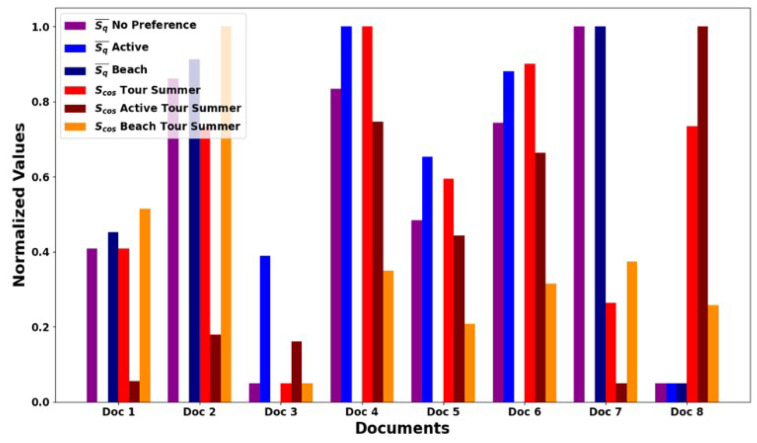
Comparative analysis of relevance metrics for synthetic documents presented in Table 1.

**Figure 10:**
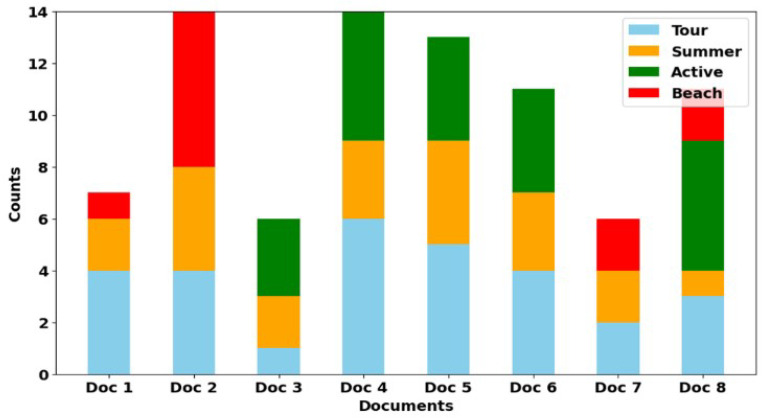
Keyword occurrence frequencies in the Documents.

The core results for our algorithm are summarized in Figure 9 and Figure 10. Figure 9 shows the normalized values of the relevance metrics, ${\bar{S}}_{q}$, and cosine similarity, $S_{\cos}$, for different user queries and preferences across eight documents from Table 1. In Figure 9, we explore metric (18) with W=7 obtained from Figure 6.

Each bar in Figure 9 represents one measure for the document to evaluate and compare the effectiveness of different ranking algorithms. The cosine similarity of $S_{\cos}$ was calculated for three queries $|\psi_{Q}\rangle$: *“Summer Tour”*, *“Summer Active Tour”*, and *“Summer Beach Tour”*, respectively. The purple bars (${\bar{S}}_{q}$, no preference) in Figure 9 demonstrate the relevance of documents regardless of the user’s specific interests. The blue and light blue bars show the values of ${\bar{S}}_{q}$ for interests *“Active”* and *“Beach”*, respectively. This demonstrates how the relevance is changing depending on the specifics of the query. The red, orange, and yellow bars show the cosine similarity values of $S_{cos}$ for the general query *“Tour Summer”* and for queries with the interests *“Active”* and *“Beach”*, respectively.

Figure 10 establishes the results of the statistical analysis of the text, counting the number of keywords in each document, which allows for assessing their lexical diversity and relevance to the query. In Figure 10, we plot a histogram showing the frequency distribution of keywords in eight synthetic documents. The colors in the bars indicate keywords, and each bar corresponds to a separate document. The different levels of the bars provide a quantitative representation of the lexical composition of each text by indicating how many times each word occurs in the corresponding document. Document 3 stands out because of its focus on outdoor activities, which is confirmed by the data shown in Figure 9 and Figure 10, respectively. Figure 10 demonstrates that the keyword *“Active”* occurs more frequently in Document 3 than other keywords. Thus, the number of references to the word *“Active”* in this document significantly exceeds the number of references to *“Tour”* and *“Summer”*, which corresponds to the topic of outdoor activities. At the same time, Figure 6 shows how the $S_{q}$ metric changes as the HAL window size increases for the *“Active”* and *“Beach”* interests. For the “Active” interest, $S_{q}$ starts higher for smaller window sizes.

Both metrics ${\bar{S}}_{q}$ and $S_{cos}$ provide important information about the relevance of documents to queries, but they can give different results depending on the structure of the text and the specifics of the query. For example, Document 4 “Bicycle tour in Tuscany” and Document 5 “Rock climbing in Colorado” show high values of both metrics in the context of *“Active”* interest, which indicates their attractiveness for queries related to outdoor activities. On the other hand, different user interests (*“Active”* and *“Beach”*) can significantly affect the relevance and ranking of documents. For example, the document “Vacation by the Sea” has a high value of ${\bar{S}}_{q}$ for interest *“Beach”*, which indicates its high specialization in this topic.

When comparing relevance metrics for document retrieval, our algorithm based on the Bell-like test parameter, ${\bar{S}}_{q}$, offers distinct advantages over traditional TF–IDF approaches, especially when analyzing documents with semantically rich content. To illustrate this, consider Document 5, entitled “Rock Climbing in Colorado”, which contains detailed descriptions of climbing routes, safety tips, and seasonal recommendations. The TF–IDF method, which emphasizes word frequency and document uniqueness, may not fully capture the thematic depth and nuanced content associated with specialized activities such as rock climbing. It tends to miss the contextual and relational nuances of terms that are critical in such specialized fields.

Conversely, the ${\bar{S}}_{q}$ metric, derived from the quantum-inspired Bell-like test, evaluates both the presence of keywords and their semantic interconnectedness within the document context. For instance, while the TF–IDF value might be high for generic terms like “Tour” and “Summer” found in the document, it does not effectively link them to the specialized context of “Active” outdoor pursuits integral to rock climbing. The ${\bar{S}}_{q}$ metric, however, identifies and enhances the relevance of connections between terms such as “Active”, “Climbing”, and “Safety”, which are crucial for users interested in rock climbing. Moreover, the analysis of Document 5 using ${\bar{S}}_{q}$ revealed a stronger alignment with user queries related to active tourism, such as “Active Tour in Colorado”. This alignment is evidenced by higher relevance scores in ${\bar{S}}_{q}$ compared to those from TF–IDF, particularly when queries include contextually rich terms associated with climbing. This demonstrates the ability of ${\bar{S}}_{q}$ to adapt to the semantic depths of the text, offering a more tailored and insightful retrieval performance. The comparison underscores the potential of the Bell-like test-based ${\bar{S}}_{q}$ metric to provide a more nuanced and context-aware retrieval mechanism in information systems, especially for texts where specialized knowledge and thematic accuracy are paramount. This highlights the inadequacies of traditional methods like TF–IDF in handling complex user needs and semantic richness, advocating for a shift towards more sophisticated, quantum-inspired algorithms in information retrieval systems.

Thus, the ${\bar{S}}_{q}$ metric is better than $S_{cos}$ in several key aspects of textual data analysis, especially when it is important to consider the contextual and semantic relationships between words and not just their superficial occurrence in texts. The peculiarity of ${\bar{S}}_{q}$, which includes taking into account changes in relevance with different sizes of context windows, allows for adapting the analysis to different styles and structures of texts. ${\bar{S}}_{q}$ can reveal these hidden relationships in situations where the text includes unique relationships between topics that are not obvious from simple word frequency.

## 6. Conclusions

In conclusion, we propose a quantum-inspired algorithm that uses heuristic Bell-like testing for document search, retrieval, and ranking, see Figure 1. Our approach considers Bell-like parameter $S_{q}$ for two-word queries in different (mutually exclusive) contexts and various user preferences. We explore the Hilbert space for describing the vector of documents and words. In particular, two words coupled via query are established in the 2D Hilbert space in different frames. This simple geometry provides a known semantics identification of two words by means of the angle (*p* parameter) between the vectors of the words. At the same time, the non-commutativity of query operators in the quantum approach to document search and ranking means detecting stronger (quantum) correlations between semantic units that classical text processing methods cannot identify. We use the HAL approach for word and document vector specification. The Bell-like parameter clearly exhibits a query order effect for two-word queries. In particular, to verify the proposed algorithm, we use a dataset of synthetic advertising text documents (see Table 1) from travel agencies generated by the OpenAI GPT-4 model. By scanning documents with HAL window sizes ranging from 10 to 80 words, we were able to demonstrate that the “entanglement” in two-word document search and retrieval can be recognized as the frequent occurrence of two words in incompatible query contexts. We have found that the user preferences and word ordering in the query play a significant role for relatively small sizes of the HAL window. For practical purposes, we suggest a new parameter ${\bar{S}}_{q}$ that considers a Bell-like parameter averaged over all HAL window sizes. This parameter allows us to evaluate the statistical properties of the texts in a new way. In particular, the comparison with the cosine similarity metrics demonstrates key advantages of our approach based on the user-enforced contextual and semantic relationships between words and not just their superficial occurrence in texts. In future, we are going to implement a machine learning algorithm to investigate some specific features of users and improve phase ϕ. In this case, it will be fruitful to introduce phase factors in query operators that can modify Bell-like parameter implementation in different contexts. We hope to implement adaptive control of the phases (user preferences) for the algorithm in Figure 1, which will help us improve the quality of document retrieval and ranking in this case.

## Figures and Tables

**Table 1:** Titles of the synthetic texts of documents analyzed in this work. The texts are given in Supplementary Materials.

Document No.	Title
1	Discover Paradise on Earth—Maldives
2	Explore the Pearl of the Adriatic—Croatia
3	Active Adventures in Patagonia
4	Bicycle Tour in Tuscany
5	Rock Climbing in Colorado
6	Hiking Excursions
7	Vacation by the Ocean
8	Outdoor Adventures

## Data Availability

All data used during this study are available within the article.

## Supplementary Materials

The following supporting information can be downloaded at https://www.mdpi.com/article/10.3390/e26100862/s1.

## Abbreviations

The following abbreviations are used in this manuscript:

AIA	Artificial Intelligence Agent
DM	Decision Making
CHSH	Clauser–Horne–Shimony–Holt
EPR	Einstein–Podolsky–Rosen
H	Hilbert Space
HAL	Hyperspace Analogue to Language
IR	Information Retrieving
LSA	Latent Semantic Analysis
NIA	Natural Intelligence Agent
