# Supplementary material for: Quantum Approach for Contextual Search, Retrieval, and Ranking of Classical Information
Source: Entropy (Basel). 2024 Oct 13;26(10):862. doi: 10.3390/e26100862 (PMC11508094; doi:10.3390/e26100862)
Supplement: Supplementary file 1 [file entropy-26-00862-s001.zip › entropy-3232700-supplementary.pdf]

Supplementary Material for Quantum approach for contextual search, retrieval, and ranking of classical information

S1 Algorithms

Algorithm S 1 Normalization and Projection Calculation

Normalization of each vector to a unit length:
$|\Psi\rangle \leftarrow \frac{1}{\sqrt{{}_D\langle\Psi|\Psi\rangle_D}}|\Psi\rangle_D$
$|u\rangle \leftarrow \frac{1}{\sqrt{\langle u|u\rangle}}|u\rangle$
$|u_+\rangle \leftarrow \frac{1}{\sqrt{\langle w_A|w_A\rangle}}|w_A\rangle$
$|v_+\rangle \leftarrow \frac{1}{\sqrt{\langle w_B|w_B\rangle}}|w_B\rangle$
Compute orthogonal bases using the Gram-Schmidt process:
$|u_-\rangle \leftarrow \mathrm{GramSchmidt}(|u_+\rangle, |w_B\rangle)$
$|v_-\rangle \leftarrow \mathrm{GramSchmidt}(|v_+\rangle, |w_A\rangle)$
Calculation of the document vector projections on the new bases:
$a \leftarrow \frac{\langle u_+|\Psi\rangle}{\sqrt{\langle u_+|\Psi\rangle^2+\langle u_-|\Psi\rangle^2}}$
$b \leftarrow \frac{\langle u_-|\Psi\rangle}{\sqrt{\langle u_+|\Psi\rangle^2+\langle u_-|\Psi\rangle^2}}$
$c \leftarrow \frac{\langle v_+|\Psi\rangle}{\sqrt{\langle v_+|\Psi\rangle^2+\langle v_-|\Psi\rangle^2}}$
$d \leftarrow \frac{\langle v_-|\Psi\rangle}{\sqrt{\langle v_+|\Psi\rangle^2+\langle v_-|\Psi\rangle^2}}$

S2 Dataset of synthetic texts

S2.1 Discover Paradise on Earth - the Maldives!

Imagine endless pristine white beaches surrounded by the crystal-clear waters of the Indian Ocean. Our exclusive Maldives tour promises not just relaxation on luxurious beaches, but also the opportunity to enjoy the underwater world by snorkeling or diving. Book your vacation with us and recharge with the beauty of this tropical paradise!

Summer is the time for new adventures and unforgettable experiences! Embark on our specially organized tour to one of the most picturesque beaches in the world. Here you will find perfect comfort: soft sand, warm sea, and first-class service. Our tour will provide you with everything you need for carefree time on the shore. Allow yourself to relax and recharge under the gentle rays of the sun!

Algorithm S 2 Calculate HAL Matrix

```
words ← number of words in text
i_0 ← 0
HAL ← 0
while i_0 < len(words) do
    LW ← index(words[i])
    RW ← index(words[i+j])
    j ← 1
    while j < len(words) − i_0 do
        HAL[LW][RW] ← HAL[LW][RW] + WS
        j ← j + 1
    end while
    i_0 ← i_0 + 1
end while
```

Algorithm S 3 TF-IDF Calculation

```
procedure ComputeTFIDF(documents)
    N ← length of documents
    df ← initialize dictionary to store document frequencies
    tf ← initialize list to store term frequencies for each document
    for d ∈ documents do
        tf_d ← initialize dictionary
        terms ← extract terms from d
        for t ∈ terms do
            if t ∈ tf_d then
                tf_d[t] ← tf_d[t] + 1
            else
                tf_d[t] ← 1
            end if
        end for
        tf.append(tf_d)
        unique_terms ← set of unique terms in tf_d
        for t ∈ unique_terms do
            if t ∈ df then
                df[t] ← df[t] + 1
            else
                df[t] ← 1
            end if
        end for
    end for
    tfidf ← initialize list of dictionaries for tf-idf scores
    for i ∈ range(N) do
        tfidf_d ← initialize dictionary
        terms_d ← tf[i]
        for t ∈ terms_d do
            tf_score ← terms_d[t] / Σ_{k∈terms_d} terms_d[k]
            idf_score ← log(N / df[t])
            tfidf_d[t] ← tf_score × idf_score
        end for
        tfidf.append(tfidf_d)
    end for
    return tfidf
end procedure
```

Choosing our tour, you will immerse yourself in an atmosphere of complete relaxation and comfort. We offer accommodation in the best frontline hotels, where each morning begins with a mesmerizing view of the endless sea and the sound of waves breaking the silence of the morning breeze. Our hotels are equipped with all modern amenities you could wish for during a beach vacation, including spa centers, open pools, seafood restaurants, and rooftop bars with panoramic views of the coastline.

Your day will be filled with a variety of activities of your choice. Start with morning yoga on the beach, where open-air meditative practices will help you achieve harmony of soul and body. Then, you might want to try surfing or test your skills in windsurfing, guided by experienced instructors. For those who prefer more peaceful activities, we offer kayaking or pedalo boating, so you can enjoy the beauty of the sea at your own pace.

Lunches and dinners are included in the tour price and represent a true culinary delight. Our chefs prepare exquisite dishes from the freshest seafood, selected every morning. You can enjoy candlelit dinners on the seaside, where the warm evening air and starry sky create a unique atmosphere of romance and relaxation.

For those seeking more adventure, we organize excursions to the region's historical and cultural landmarks. Discover ancient ruins, mysterious castles, and cozy harbor towns with their unique charm and traditions. Every evening, entertainment events are held on the beach, including live music, dances, and open-air movie screenings.

S2.2 Explore the Pearl of the Adriatic - Croatia!

Join our tour to Croatia, where the sun, sea, and sand blend with unique culture and delicious local cuisine. Travel through picturesque bays, enjoying sea swims and sunbathing on secluded beaches. This tour is ideal for those who dream of the perfect beach vacation with a taste of adventure.

Ready for a summer adventure? Choose our tour to exotic beaches where comfortable relaxation amid heavenly landscapes awaits you. Crystal-clear sea, pristine white sand, and cozy loungers—all are designed for your perfect summer. We've ensured your vacation is as relaxing as possible, from spa treatments on the beach to gourmet candlelit dinners. Relax comfortably and enjoy every moment!

Our tour offers you a unique opportunity to experience the full charm of summer relaxation on the coast. Forget the hustle and bustle of the city and dive into an atmosphere of complete tranquility and relaxation. Each morning begins with a beautiful sunrise, which you can watch from the balcony of your room overlooking the sea. Breakfast will be served right on your terrace, allowing you to enjoy the first rays of sun and a light sea breeze.

Continue your day with a swim in the crystal-clear water or relax on a lounger with your favorite book. Our beach is equipped with all the essentials for your convenience: umbrellas, towels, and comfortable loungers are always at your disposal. Various water sports such as snorkeling, water skiing, or paragliding are available to add adrenaline and fun to your vacation.

For those who prefer quieter activities, yoga and meditation on the beach are offered. These sessions are conducted by experienced instructors and will help you achieve inner harmony and peace. As the sun begins to set, our beach transforms into a venue for romantic candlelit dinners, where you can enjoy exquisite dishes of local and international cuisine.

After dinner, we invite you to stroll along the shore under a starry sky. Enjoy the warm evening air and the sound of the waves, which will carry away all your worries. Our program also includes various evening events and shows, making every evening truly unforgettable.

We care about every detail to provide you with an exceptional level of comfort and relaxation. By choosing our tour, you're not just opting for a beach holiday but a complete journey into a summer paradise, where each day is filled with joy, tranquility, and beauty.

S2.3 Active Adventures in Patagonia

Are you ready for thrilling and active summer adventures in pristine wilderness? Our exclusive tour to Patagonia invites you on an unforgettable journey filled with unique trekking trails, kayaking among majestic glaciers, and encounters with rare species of flora and fauna. This active holiday will not only allow you to explore the most picturesque corners of South America but also give you the opportunity to feel like a true part of nature. Prepare for days full of adventures and discoveries, where every moment is imbued with a sense of freedom and awe at the surrounding beauty. Ahead of you are dynamic routes

along winding paths, beside rivers, and through alpine meadows, offering unprecedented opportunities for photography and wildlife observation.

Patagonia is a place where every step unveils new breathtaking summer views and perspectives, and the fresh mountain air energizes you for the entire day. You will traverse routes through dense forests, rocky plateaus, and snow-capped peaks, where you can enjoy crystal-clear skies and the unique tranquility of remote areas. Discover new horizons on a journey that offers not just active recreation but also opportunities for meditation and thoughtful solitude in the embrace of nature. Give yourself time to slow down and savor every moment spent amidst monumental mountains and icy fields.

Join us on this amazing trip and discover the wild beauty of Patagonia. We promise that this adventure will stay with you for a lifetime, leaving indelible impressions and memories of unparalleled natural beauty. An expedition to Patagonia promises to be not only a journey across the world but also a journey to oneself, where everyone finds something valuable and meaningful. Join our journey and become part of this unique expedition to one of the most thrilling places on the planet. Ahead of you are wonders of nature, warm encounters with locals, and many unforgettable moments that will warm you with memories for many years. Dive into this whirlwind of adventures, where each day promises new discoveries, encounters with unique animals like pumas and Andean condors, and challenges that will turn your journey into a true test of spirit and body.

S2.4 Bicycle Tour in Tuscany!

Explore the beauty of Tuscany by cycling through the green hills and vineyards of this region of Italy. This tour is suitable for both beginners and experienced cyclists. You'll meet local residents, taste the finest Tuscan wines, and enjoy breathtaking landscapes. Have an active and enriching break!

Looking for an unforgettable summer vacation? Our active tour offers you a journey full of adventures and new experiences. Go hiking on scenic trails, explore hidden corners of nature, and fully enjoy the beauty of the summer landscape. This tour is ideal for those who want to combine active recreation with comfort and safety. Forget routine and allow yourself a true adventure!

We invite you to dive into the world of active tourism, where every day turns into an exciting adventure. Start the morning with yoga by the lake, where silence and tranquility fill your consciousness, preparing you for new challenges. Then, head out on bike rides along mountain slopes, discovering magnificent views and enjoying the fresh mountain air. Your route will be planned so that you can explore both well-known attractions and secluded trails accessible only to the most daring travelers.

After lunch in a local village, where you can try traditional dishes and learn more about the region's culture, it's time for kayaking or rafting. The rivers in this area are known for their turbulent currents, which promise not only to test your bravery but also to provide unforgettable emotions. And of course, your evenings will also be filled with coziness and comfort: enjoy dinners by the fire, exchange stories with new friends, and plan future adventures under the starry sky.

Our tour company cares about your comfort at every stage of the journey. We provide all necessary equipment for activities, arrange transportation between locations, and ensure safety with experienced guides and instructors. Every aspect of your tour is carefully thought out so that you can enjoy every moment of your vacation without worries.

Join our summer active tour, and you'll see what real relaxation is. Discover not only new places but also new sides of yourself, gain inspiration and energy that will stay with you long after you return home. Allow yourself to discover the world of true adventures this summer!

S2.5 Rock Climbing in Colorado!

Join our exciting tour in Colorado, where you can conquer the peaks of the Rocky Mountains. Regardless of your experience, we offer routes of various difficulties, guided by experienced instructors. Feel the adrenaline and enjoy the incredible views from the tops that will be memorable.

Planning your summer vacation? Choose our unique tour for an active journey! Immerse yourself in the world of water sports, mountain biking, and rock climbing. Each day of our tour promises new challenges and thrilling moments that will stay in your memory forever. Turn your summer into a series of exciting adventures, where each route intensifies your desire to explore and actively relax. Don't miss the chance to make this summer truly unforgettable!

Discover the splendor of crystal-clear lakes, mountain rivers, and wild nature during our specially designed expeditions. Start the day with a morning swim in a quiet bay, where you can enjoy tranquility

and gather strength before the upcoming challenges. Then head out for an exhilarating descent on a mountain bike through the serpentines of mountain roads, where each turn hides breathtaking views and new opportunities for photography.

After lunch, enjoy an hour of rest in the shade of dense forests, where you can relax or, if the desire to explore persists, go on a short hike to a recently discovered waterfall. In the evening, enjoy rock climbing under the guidance of our experienced instructors. This is the perfect opportunity to test your strength and see the world from a bird's eye view.

Your evenings will be as eventful as your days. Dinner under the stars, aromatic local coffee, and cozy gatherings by the campfire will give you time to connect with like-minded individuals and share impressions of the day. Every element of our tour is designed so that you can relax both body and soul, recharge with new ideas and emotions.

We take care to ensure that your active vacation is safe and comfortable. All our routes are carefully checked, and the equipment meets the highest standards of quality. Our guides and instructors are professionals who not only excel in their field but are also eager to share their knowledge and experience.

Join our active tour and experience what true adventure is. Traveling with us is not just moving from point A to point B, it's an opportunity to see the world differently, explore your limits, and meet people who share your passion for active leisure. Book your spot now and make this summer truly unforgettable!

S2.6 Hiking Excursions

Looking for an unforgettable summer vacation? Our active tour offers you a journey full of adventure and new experiences. Embark on hiking excursions along scenic trails, explore hidden corners of nature, and fully enjoy the beauty of the summer landscape. This tour is ideal for those who want to combine active leisure with comfort and safety. Forget the routine and allow yourself a real adventure!

We invite you to dive into the world of active tourism, where each day turns into an exhilarating adventure. Start your morning with yoga by the lake, where tranquility and peace fill your consciousness, preparing you for new challenges. Then, head out on mountain bike rides along mountain slopes, discovering magnificent views and enjoying the fresh mountain air. Your route will be planned to allow you to explore both well-known landmarks and secluded trails, accessible only to the most daring travelers.

After lunch in a local village, where you can try traditional dishes and learn more about the region's culture, it will be time for kayaking or rafting. The rivers in this area are known for their turbulent currents, which promise not only to test your bravery but also to provide unforgettable emotions. And of course, your evenings will also be filled with coziness and comfort: enjoy dinners by the campfire, exchange stories with new friends, and plan future adventures under the starry sky.

Our tour company cares about your comfort at every stage of the journey. We provide all the necessary equipment for activities, arrange transportation between locations, and ensure safety with experienced guides and instructors. Every aspect of your tour is carefully thought out so you can enjoy every moment of your vacation worry-free.

Join our summer active tour, and you will see what real relaxation is. Discover not only new places but also new aspects of yourself, find inspiration and energy that will stay with you long after you return home. Allow yourself to open up to the world of true adventures this summer!

S2.7 Vacation by the Ocean

Dreaming of a summer vacation by the ocean? Our tour offers you a journey to the world's best beaches this summer, where everyone will find something for themselves. Enjoy comfortable relaxation in luxurious bungalows right by the water, explore the underwater world with scuba diving or simply relax under an umbrella, inhaling the scent of the sea. We take care of all the details of your trip, so you can effortlessly enjoy the sun and sea. This summer tour will be your perfect way to unwind and forget about everyday hustle and bustle.

Your journey begins with a comfortable flight to one of the most scenic places on Earth, where the warm waters of the tropical ocean meet pristine sandy beaches. Upon arrival, you will be transferred to a luxurious resort, where you will be accommodated in spacious bungalows with ocean views. Each bungalow is equipped with the latest in technology and design, offering not only seclusion and tranquility but also exclusive beach access.

Professional instructors will help you master diving or snorkeling so you can explore the rich underwater world of coral reefs inhabited by diverse marine life. If you prefer more tranquil activities, we offer yoga sessions at dawn by the sea or romantic walks at sunset, when the sky plays with shades of red and orange.

Our culinary offerings cover a wide range of choices: from fresh seafood caught near the shore to exotic fruits grown on local plantations. Try unique local dishes at one of our open-air restaurants or enjoy an evening cocktail at the poolside bar, where music and waves create a unique atmosphere.

For your convenience, the resort offers various services, including full-service spas with a range of treatments from massages to skincare and hair care, helping you achieve complete relaxation and rejuvenation.

In the evenings, the resort comes alive with live performances by local artists and themed beach parties. It's the perfect opportunity to dance barefoot on the sand under the starry sky, ending each day of your vacation with bright and memorable moments.

S2.8 Outdoor Adventures

Are you drawn to active summer vacations and outdoor adventures? Discover a unique tour that offers not only dynamic routes for trekking and kayaking but also the chance to enjoy pristine beaches with crystal-clear water. Join us on a journey that will take you away from the daily hustle and bustle into a world of adventure and discovery!

Our active tour features routes that pass through scenic forests, mountain ranges, and along rivers, where each step unveils new thrilling vistas. Besides trekking, you'll experience kayaking on turbulent rivers and peaceful strolls along beaches, where you can fully feel a connection with nature. This tour is perfect for those seeking an active holiday with elements of tranquil nature experiences.

Discover new places and challenge yourself by participating in our active tour. We promise that this adventure will leave indelible impressions in your memory, filled with excitement from discoveries and joy from interacting with nature. Join us and experience all the delights of active recreation in amazing natural settings, where everyone will find something special for themselves!
